# In-Flight Observation and Surface Oxidation Modification of Tin Oxide Nanoparticles for Gas Sensing Applications

**DOI:** 10.1021/acsanm.5c00144

**Published:** 2025-03-18

**Authors:** Calle Preger, Linnéa Jönsson, Pau Ternero, Mehran Sedrpooshan, Marie Bermeo, Antti Kivimäki, Noelle Walsh, Maria E. Messing, Axel Christian Eriksson, Jenny Rissler

**Affiliations:** 1 MAX IV Laboratory, Lund University, Box 118, Lund 221 00, Sweden; 2 Ergonomics and Aerosol Technology, Lund University, Box 118, Lund 221 00, Sweden; 3 NanoLund, Lund University, Box 118, Lund 221 00, Sweden; 4 Solid State Physics, Lund University, Box 118, Lund 221 00, Sweden; 5 Synchrotron Radiation Research, Lund University, Box 118, Lund 221 00, Sweden; 6 RISE Research Institutes of Sweden, Scheelevägen 17, Lund 223 70, Sweden

**Keywords:** tin oxide, nanoparticles, oxidation, reduction, in-flight, aerodynamic lens, aerosol sample-delivery system, catalysis

## Abstract

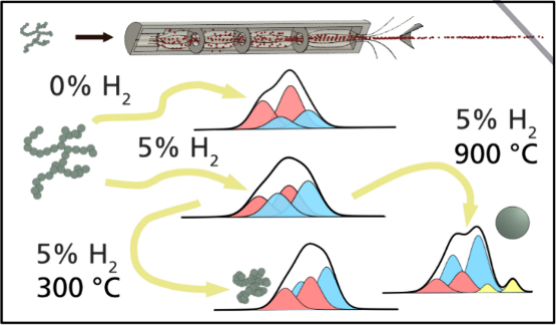

Metal oxide nanoparticles are essential in various applications, and the synthesis through gas-phase generation methods offers a rapid and reliable pathway for nanoparticle production. Yet achieving precise control over their formation remains challenging due to the complex nature of oxidation processes. While bulk oxidation states can be assessed via off-line measurements, the dynamic nature of surface oxidation is more difficult to monitor and optimize in real time. Here, we investigate the surface oxidation state of unsupported tin oxide nanoparticles using an aerosol sample-delivery system and in-flight X-ray photoelectron spectroscopy. This powerful method allows the continuous monitoring of the surface oxidation of the gas-phase generated nanoparticles in real time, avoiding uncertainties associated with postcollection alterations. Tin oxide nanoparticles are widely used in gas sensing and catalytic applications, where the surface oxide layer plays a crucial role in determining their performance. Our findings demonstrate how the surface oxidation state of the free-flying particles can be controlled by adjusting the carrier gas composition, in-flight heating temperature, and particle composition. Specifically, the surface oxides of tin are partially reduced when heated in a slightly reducing atmosphere, and the reduction is further enhanced by forming mixed tin–gold nanoparticles. While previous studies on metal oxide nanoparticles have focused predominantly on bulk properties or off-line analysis, this study employs real-time in-flight X-ray photoelectron spectroscopy to investigate details of the surface oxidation state. Understanding the surface oxidation of metal oxide nanoparticles is essential to optimize processes, such as in-flight coating or subsequent deposition into a protective environment. This approach enables the exploration of direct correlations between generation conditions and surface properties, providing valuable insights into optimizing gas-phase nanoparticle synthesis.

## 1 Introduction

The gas-phase generation of metal nanoparticles using aerosol methods offers a rapid and reliable pathway for producing nanoparticles suited to a wide range of applications.^1,2^ Aerosol generation methods can produce diverse material combinations, including monometallic, bimetallic,^3,4^ and multielemental^5,6^ particles, as well as metal–metal oxide combinations.^7−9^ Metal oxide nanoparticles are a promising class of materials for a large range of technical applications due to their chemical stability and diverse tunable properties. These applications span various fields, including electrical, optical, magnetic, catalytic, and photochemical domains.^10−12^

Tin (Sn) metal oxide nanoparticles are notable due to the variation of their properties (electronic properties, in particular) with their oxidation state. SnO_2_, a stable oxide, is an n-type semiconductor with a wide band gap and excellent thermal stability, making it widely used in gas sensors and catalytic reactions.^10,13−15^ In contrast, SnO, while also a wide band gap semiconductor, is far less useful due to its high reactivity. Small traces of O_2_ cause SnO to convert into SnO_2_, and in the absence of oxygen, SnO undergoes disproportionation, decomposing into Sn.^16^ Sn nanoparticles, when alloyed with other metals, have also shown potential for catalytic applications.^17,18^

The properties of Sn metal oxide nanoparticles are highly dependent on their chemical surface composition, and varying the oxidation state can change the functionality of the nanoparticles. However, understanding and controlling the highly dynamic changes occurring at the surface of aerosol particles in response to generation conditions, such as applied temperature and gas composition, and being able to fine-tune the nanoparticles’ surfaces for specific purposes present significant challenges. One challenge is that to study surface oxidation, tools for analyzing the surface oxides directly during the generation process are needed. Traditional off-line characterization methods are limited in their ability to precisely capture these surface alterations in real time. Therefore, achieving a comprehensive understanding of the nanoparticles requires adopting novel methodologies for analyses.

The development of the aerodynamic lens^19^ for mass spectrometry studies on aerosols^20^ enabled online, or in-flight, spectroscopy measurements at synchrotron facilities.^21−23^ In-flight X-ray photoelectron spectroscopy (XPS) has been used to investigate various aspects of nanoparticle characteristics at the particles’ surface such as the oxidation state,^24,25^ electronic properties,^26,27^ and water adsorption.^28^ However, a significant gap remains in understanding and exploring how external factors, such as gas composition and temperature, affect the surface oxidation of metal oxide nanoparticles under real-time atmospheric pressure conditions.

In this work, in-flight XPS was used to investigate the oxidation and reduction of aerosol Sn and Sn–Au nanoparticles, focusing on how the Sn surface oxidation state responds to carrier gas composition, in-flight heating temperature, and the presence of mixing elements. We demonstrate that the properties of the surface oxide are indeed sensitive to these applied conditions, more specifically that the surface structure of Sn can be partially reduced when heated in a reducing environment, and that this process is enhanced for mixed Sn–Au nanoparticles. The surface oxidation of Sn nanoparticles significantly influences their gas sensing and catalytic performance. Understanding and controlling this oxidation process enable the synthesis of more optimized nanoparticle-based structures. As most previous investigations have been focused on off-line bulk properties, this investigation adds important knowledge for advancing and optimizing the gas-phase production of metal oxide nanoparticles by studying the surface properties in real time by in-flight XPS, also showcasing the possibilities of applying a new state-of-the-art characterization technique.

## 2 Methods

### 2.1 Aerosol Sn Nanoparticle Synthesis

**Figure 1:**
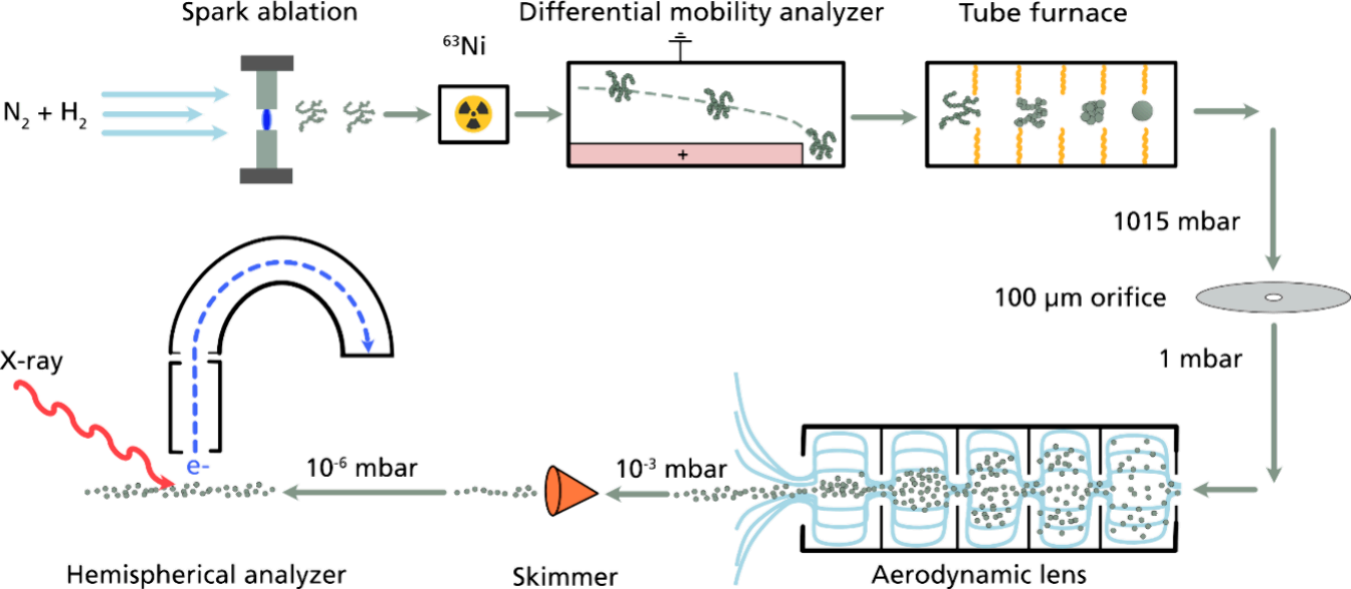
Experimental setup of the aerosol nanoparticle generation by spark ablation and subsequent transfer to the aerosol sample-delivery system (ASDS). The nanoparticles are generated by spark ablation in a continuous gas flow at atmospheric pressure. The differential mobility analyzer allows for size selection, and the agglomerates are reshaped in the tube furnace. The aerosol particles are transferred to the ASDS via a 100 μm flow limiting orifice. The particles are thereafter collimated into a narrow beam of particles in the aerodynamic lens before entering the interaction chamber, where the photon beam interacts with the particles, causing electrons to be emitted and detected.

An overview of the experimental setup is shown in Figure 1. Engineered nanoparticles (Sn and Sn–Au) were generated by spark ablation.^29^ Spark ablation is an aerosol nanoparticle generation method that produces a high concentration of nanoparticles typically in a continuous gas flow at atmospheric pressure.^30^ Two gas lines containing N_2_ and N_2_ with 5% H_2_ provided the carrier gas (1.68 L/min). The H_2_ concentration in the carrier gas was tuned from 0 to 5% H_2_ by changing the flow rate ratio between the two gas lines. Two Sn electrodes (3 mm in diameter) were charged to create sparks that ablated material from their surfaces. For the generation of Sn–Au nanoparticles, one of the Sn electrodes was replaced by an Au rod (3 mm in diameter). The metal vapor that is produced by the sparks nucleates to sub-10 nm primary particles that diffuse, collide, and coalesce to form larger agglomerates. The agglomerates obtain a known charge distribution by passing through a ^63^Ni bipolar charger.^31^ In the setup used, the agglomerates either passed through a differential mobility analyzer (DMA) (TSI Model 3081) for in-flight size-selection or bypassed the DMA. The DMA operated with a 1:6 flow ratio between the carrier gas and the sheath gas. The sheath gas was composed of pure N_2_ regardless of the carrier gas composition. The agglomerates thereafter passed through a tube furnace (20–1300 °C) before depositing for off-line analysis or were directed to the aerosol sample-delivery system (ASDS)^23^ for in-flight XPS analysis. The nanoparticles used for off-line analysis were sampled onto copper grids using an electrostatic precipitator for transmission electron microscopy (TEM) analysis using a JEOL 3000F field emission electron microscope.

### 2.2 X-ray Photoelectron Spectroscopy

The ASDS^23^ was installed at the gas-phase end station (GPES)^32^ of the FinEstBeAMS beamline^33^ at MAX IV Laboratory (Lund, Sweden). The ASDS operates by transporting aerosol particles from atmospheric pressure to a vacuum chamber for in-flight XPS measurements in the shape of a narrow and collimated particle beam. The aerosol was introduced to the ASDS through a 100 μm critical orifice (Figure 1), resulting in a constant mass flow rate of 0.088 L/min at atmospheric pressure. Thereafter, the particles were led through a 686 mm long relaxation tube before entering the aerodynamic lens (Aerodyne PM1) with orifice dimensions that can be found in the work of Zhang and coauthors.^34^ The ASDS reduces the pressure stepwise from atmospheric pressure to 10^–6^ mbar and produces a narrow, collimated, and concentrated particle beam (<0.5 mm in diameter).^23^ The particle beam thereafter passes through a 1.0 mm conical skimmer separating the ASDS from the GPES at FinEstBeAMS. In the GPES, the aerosol particle beam crosses the photon beam, and the interaction between the nanoparticle beam and the X-rays.

The skimmer separating the aerodynamic lens and the interaction chamber further reduces the pressure in the interaction region to approximately 1 × 10^–6^ mbar. When the ASDS is coupled to the GPES, the SCIENTA R4000 electron analyzer of the GPES is rotated in a vertical position. To maximize the electron intensity in that direction, the radiation source of the beamline, an elliptically polarizing undulator, was set to provide vertically polarized light. The photon energy was set at 104 eV, and Sn 4d photoelectrons were collected with a kinetic energy of approximately 70 eV to achieve high surface sensitivity. At this photon energy, the FinEstBeAMS beamline offers a maximal photon flux, and the Sn 4d photoionization cross section is high. The electron inelastic mean free path for Sn at this photon energy is approximately 0.6 nm.^35^ The exit slit of the beamline was kept at 600 μm, and the spectrometer was operated with a 100 eV pass energy and a 400 μm curved slit, which provided a total instrumental energy resolution of about 75 meV. Each spectrum was acquired in 10 to 30 min depending on the signal intensity, which varied under different generation conditions.

The two Sn 4d spin–orbit components in the photoelectron spectra were fitted with a 3:2 intensity ratio and a 1.1 eV splitting. The peak position was kept as a free variable, while the binding energies of the Sn^2+^ and Sn^0^ peaks were kept at 0.7 and 2.0 eV lower, respectively, than the binding energy of the Sn^4+^ peaks.^36−38^ All peaks were fitted with a Gaussian/Lorentzian sum function using the CasaXPS software. The full width at half-maximum (fwhm) was kept in a narrow range for all peaks (0.9–1 eV for oxides, 0.5–0.6 eV for zerovalent Sn).

Since XPS measurements for unsupported nanoparticles (as in the case of the ASDS) determine the electron energies relative to the vacuum level and not the Fermi level, an energy shift corresponding to the materials’ work function (approximately 2 to 6 eV) is expected compared to the XPS reference energies reported in the literature. The binding energy scale was determined relative to the vacuum level after energy calibration using the binding energy of the outermost valence orbitals of N_2_ at 15.58 eV.^39^

## 3 Results and Discussions

### 3.1 Effect of Carrier Gas on Sn Oxidation

The surface oxidation of free unsupported Sn nanoparticles in response to various generation parameters was investigated in-flight using XPS. Sn generated nanoparticles were synthesized using different (inert and reducing) carrier gases and in-flight heating temperatures (from room temperature to 900 °C). As mentioned in the Methods section (Section 2.1), the spark ablation generated sub-10 nm particles rapidly coagulate to form larger agglomerates consisting of the primary particles as they are transported away by a carrier gas. For in-flight detection, agglomeration of the sub-10 nm particles is a requirement to ensure a sufficient concentration of particles with a mobility diameter optimal for aerodynamic lens collimation.^34^

**Figure 2:**
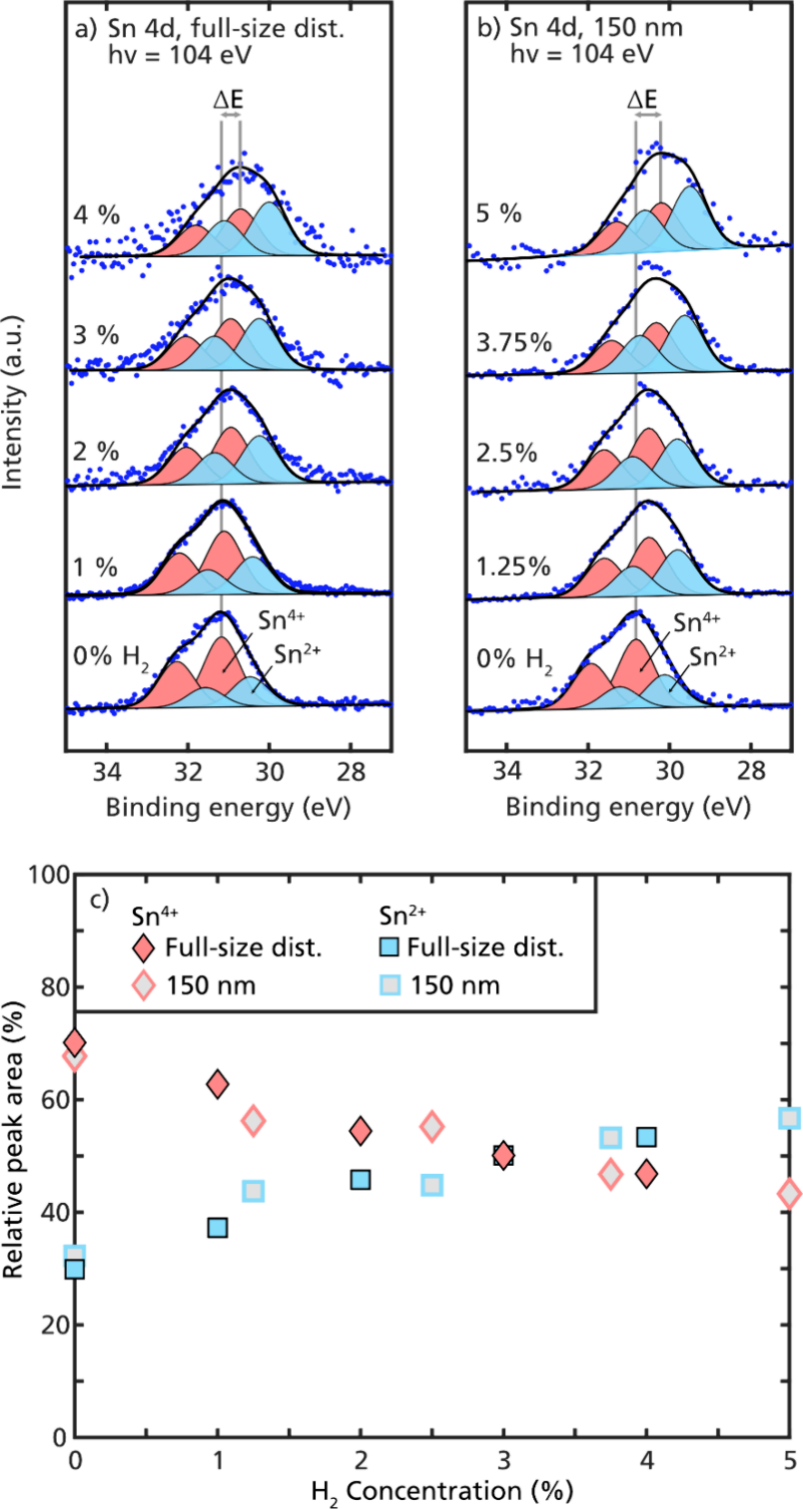
The XPS spectra for Sn nanoparticle agglomerates of the (a) full-size and (b) size-selected (150 nm) distribution with increasing H_2_concentration in the carrier gas. The blue dots are the background subtracted data points, and the black line is the sum of the fitted peaks (shaded profiles). (c) The relative peak areas of Sn^4+^(red diamonds) and Sn^2+^(blue squares) are plotted against the H_2_ concentration in the carrier gas. The size-selected and full-size distributions follow the same trend with a decreasing amount of Sn^4+^with increasing H_2_ concentration in the carrier gas.

The Sn agglomerates were initially measured without any in-flight heating-step, generated using N_2_ as the main carrier gas, but with a varied added concentration of H_2_ 0–5% to the carrier gas (Figure 2a). Similar measurements were also performed for size-selected agglomerates to evaluate the influence on the particle size and the charge distribution of the particles (Figure 2b). Despite using inert gases, small traces of oxygen are expected to be present during generation causing the nanoparticles to oxidize, as the spark ablation method is employed at atmospheric pressure.^40^ The H_2_ in the carrier gas is added to suppress this oxidation or to reduce the particles in the transportation line.

Comparing the chemical surface oxidation of the full-size distribution with size-selected 150 nm agglomerates showed a similar oxidation state in terms of the relative peak area ratio between the two fitted Sn oxides (Sn^4+^ and Sn^2+^). For both the full-size and size-selected distributions, an increasing concentration of H_2_ in the carrier gas resulted in a less oxidized surface. The shift in oxidation gradually changed when increasing the concentration of H_2_ in the carrier gas in the 0–5% range. The surface oxides shifted from approximately 75% Sn^4+^ with no H_2_ present in the carrier gas to approximately 40% Sn^4+^ with 5% H_2_ (Figure 2c). The similarities between the full-size and size-selected distributions suggest that reduction occurs in the spark ablation region rather than during transportation. This is because the carrier gas is diluted at a 1:6 ratio with pure N_2_ after passing through DMA, influenced by the sheath gas.

The determined binding energies of the Sn^4+^ and Sn^2+^ peaks were not the same for the full-size distribution as those for the size-selected particles. At 0% H_2_, the binding energy to vacuum for Sn^4+^ for the full-size distribution was determined to be 31.2 eV, whereas it was shifted to 30.8 eV for the size-selected 150 nm agglomerates (Figure 2a,b). This shift in energy can only be explained by changes in particles’ properties since no energy shift was observed for the measured background gas molecules (Figure S1). The shift in energy is attributed to changes in the particles’ electronic work function since the binding energies of these in-flight measurements were measured relative to the vacuum level and not the Fermi level.

Apart from the obvious differences in the size distribution, the main difference between the size-selected and the full-size distribution is related to the particles’ charge distribution. The DMA was operating with positive polarity, and only particles with negative charge were selected. The size-selected particles therefore carried negative charge, in contrast to the full-size distribution where the charge of most particles is expected to be zero after passing through the ^63^Ni neutralizer.^31^ This could partly explain the observed shift in energy as the electronic work function of negatively charged particles is expected to be lower than that of the neutral particles.^41^ A more detailed discussion about the observations related to the work function is found in the final section (Section 3.4) of this article.

The introduction and addition of H_2_ to the carrier gas have previously been reported to be a pathway to form unoxidized nanoparticles.^42^ However, the previous characterization has only been performed using bulk sensitive methods (e.g., X-ray diffraction) and furthermore investigated after depositing the particles to a substrate, storing, and transporting the samples to the instrument in ambient atmosphere. Here we can conclude that the surface of the nanoparticles is oxidized even when utilizing a carrier gas with 5% H_2_. It can also be concluded that this surface oxidation occurs already in the aerosol phase, at generation, and not postdeposition and that it varies with the carrier gas. Using a carrier gas with 5% H_2_ and no in-flight heat treatment, none zerovalent Sn (Sn^0^) was observed at the surface of the nanoparticles. Nevertheless, it should be underlined that our findings do not contradict the previous findings demonstrating the formation of metallic Sn nanoparticles using a carrier gas with 5% H_2,_^42^ as it is possible that zerovalent Sn is found in the bulk structure of the nanoparticle. Instead, here, we provide additional information about the surface oxidation of the aerosol nanoparticles.

### 3.2 Surface Oxidation with In-Flight Heating

To investigate how in-flight heating influences the surface oxidation of the Sn agglomerates, XPS was performed at different in-flight furnace temperatures with a carrier gas composition of 5% H_2_ in N_2_. The heating of the agglomerates occurs with a residence time in the tube furnace estimated to be around 5 s. After the tube furnace, the particles travel for a few seconds before reaching the photon beam.

**Figure 3:**
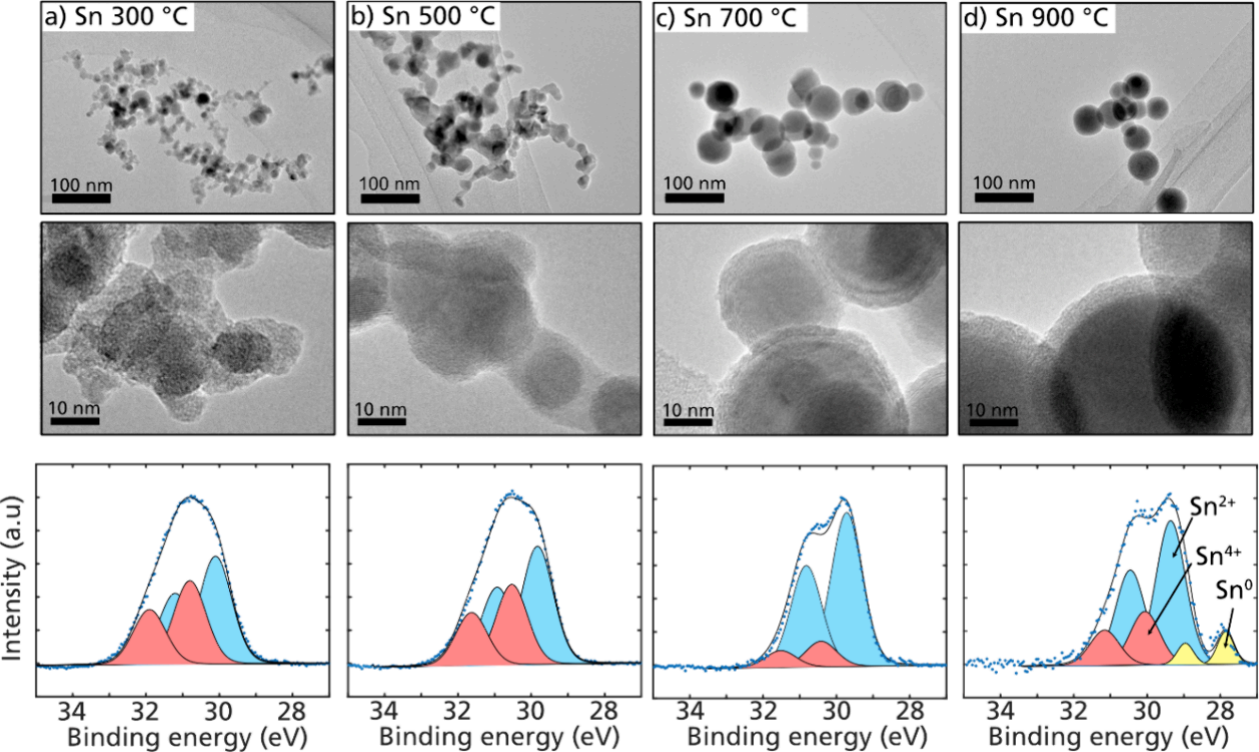
TEM images of particles generated at different in-flight heating temperatures (top and middle row) and Sn 4d photoelectron spectra recorded with the corresponding conditions (bottom row). The morphology of the nanoparticle agglomerates changes with an increased annealing temperature. At 300 and 500 °C, the agglomerates are fractal-like (a, b), whereas above 700 °C, the nanoparticles become spherical in shape (c, d). Note that the large 70 nm primary particles shown in parts c and d are deposited close to the TEM grid and do not form an aggregate in the gas. As the furnace temperature increases, the particle surface composition is reduced. At 900 °C, Sn^0^ appears on the surface of the particles.

Upon sufficient heating, the morphology of agglomerates undergoes substantial transformation, with fractal-like structures rearranging into more compact shapes due to particle sintering, driven by elevated temperatures or extended residence times in the tube furnace. This well-known process is commonly utilized to achieve spherical nanoparticles with a well-defined size in the gas flow.^43^ Sintering occurs gradually by restructuring the atoms in the necking region between two adjacent primary particles, driven by the reduction of the particles’ surface energy. The neck region grows, and the primary particle increases in size to finally transform into a dense nanoparticle. This process is visualized by the TEM images in Figure 3 (top row).

At 300 °C, the Sn agglomerates are still elongated and fractal-like, and by increasing the temperature, the agglomerates become more compact (Figure 3a–d). Above 700 °C, the nanoparticles are spherical in shape, and the sintering is completed. It should be noted that the surface structure appears to change from an amorphous structure at the lower temperatures to a more ordered shell structure at the higher temperatures (Figure 3, middle row). However, it is not possible to determine whether the shell structure in the TEM images above 700 °C also is present in the aerosol phase since we cannot conclude if this shell is formed after deposition or when exposed to the intense electron beam during TEM imaging. Although the shape of the nanoparticles does not appear to change much above 700 °C, it is evident from the XPS spectra that the surface oxidation continues to change in the aerosol phase. With increasing temperatures, the Sn^2+^ becomes more dominant, and at 900 °C, a reduction is achieved that would enable the formation of zerovalent Sn at the surface of the nanoparticles (Figure 3, bottom row).

**Figure 4:**
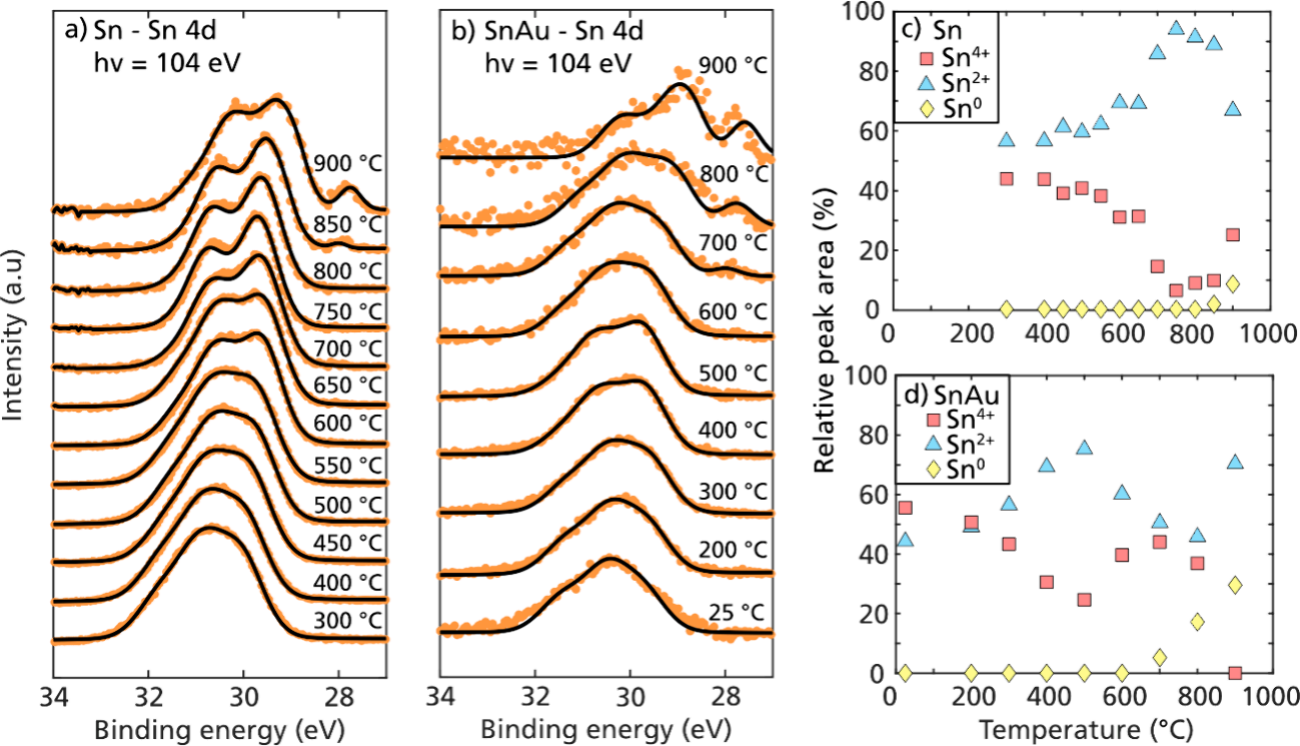
Sn 4d photoelectron spectra of (a) Sn and (b) Sn–Au with increasing in-flight heating temperatures. Orange dots are the background-subtracted data points, and black lines are the sum of the peak fitting. When the temperature increases, the spectral shape changes as the surface becomes more reduced. Above 800 °C for Sn and above 600 °C for Sn–Au, a new feature appears that can be attributed to Sn^0^. The relative peak areas of the three different components (Sn^4+^, Sn^2+^, and Sn^0^) are plotted as a function of temperature for Sn in panel c and for Sn–Au in panel d.

The influence of the in-flight heating temperature on the surface oxidation was investigated in detail by increasing the temperature in stepwise increments and observing changes in the XPS spectra. The XPS spectra, shown in Figure 4a, changed from one broad peak at 300 °C to a double-peak feature at temperatures above 550 °C, where the reduction of SnO_2_ in H_2_ is known to be favorable, with the formation of water molecules.^44^ Above 850 °C, a third doublet is observed at an even lower binding energy, which is assigned to Sn^0^.

Sn–Au nanoparticles were synthesized and analyzed by using the same experimental setup. These nanoparticles are of interest due to their catalytic properties.^18^ Furthermore, understanding how Sn surface oxidation is affected by the formation of a bimetallic structure with Au, compared with the monometallic counterpart, is important for optimizing surface oxidation. The change in elemental bulk ratio along with the detailed crystalline structure of these Sn–Au nanoparticles has been presented elsewhere.^45^ There it was shown that without any heat treatment, the synthesized Sn–Au nanoparticles had a close to 1:1 bulk atomic ratio between Sn and Au. However, when heated in-flight, the elemental ratio changed, and the amount of Sn varied with increasing temperature.^45^

The surface oxidation of nanoparticles of Sn and Sn–Au displayed a trend similar to that as a function of in-flight heating temperatures (Figure 4b). The relative peak areas from fitting of the three different doublets (Sn^4+^, Sn^2+^, and Sn^0^) are compared in Figure 4c,d for the Sn and Sn–Au particles. Although Sn and Sn–Au follow a similar trend, the reduction of the surface oxide for the Sn–Au mixture occurred at lower temperatures than those of Sn particles. For Sn at about 600 °C, the amount of Sn^2+^ starts to rise while Sn^4+^ decreases, and Sn^2+^ reaches a maximum at approximately 800 °C. Above this temperature, Sn^0^ also appears, although in low concentrations. At 800 °C, where Sn^0^ starts to form, the results from fitting the XPS distribution show a decrease in the relative amount of Sn^2+^, while Sn^4+^ increases again. For Sn–Au nanoparticles, the rise in Sn^2+^ is initiated already at 300 °C and reaches a maximum at 500 °C. Also, Sn^0^ is formed at lower temperatures for Sn–Au, and it can be observed already at 700 °C. The concentration of Sn^0^ continues to rise with increased temperature.

From these results, it is evident that mixing Sn with Au has a significant impact on the surface oxidation of Sn, as it lowers the required heating temperature to form Sn^0^ on the surface of the nanoparticles. An explanation for this appearance is that the catalytically active Au atoms act as adsorption sites for H_2_, causing them to more quickly adsorb, dissociate, and react with the oxygen on the Sn surface.^46^

### 3.3 Stoichiometric or Intermediate Oxides

The oxidation of Sn has been a topic of discussion for many years, with numerous attempts to unravel the mechanisms involved. The oxide is suggested to be formed through a Cabrera–Mott-driven reaction, with an oxidized shell separated from the metal structure.^47^ The oxide has been proposed either to be composed of separated domains of SnO and SnO_2,_^35^ or to appear in the form of a mixture of SnO and SnO_2._^48^ Also, the existence of intermediate oxides in the structure has been proposed.^49−51^ The generation of aerosol Sn nanoparticles has previously been shown to form nonstoichiometric intermediate oxide (SnO_*x*_) in the gas phase^52^ with the degree of oxidation varied from *x* = 1.2 to 1.8. The formation of the nonstoichiometric intermediate oxide in the gas phase has also been shown to suppress further oxidation to form the stoichiometric stable oxide (SnO_2_).^53^ For the surface oxides of Sn, more evidence points toward the formation of intermediate oxides rather than stoichiometric stable oxides.^16^ Often, the stoichiometric Sn-oxides are determined in the analysis of XPS spectra in the literature; however, there are exceptions where the formation of intermediate oxides is argued.^54^

The spectral peak fitting presented so far in this manuscript has been based on the results from De Padova et al. (1994),^36^ and most publications on Sn 4d used the same approach. However, Wright et al. (2017)^38^ recently presented evidence for an alternative interpretation of the Sn 4d XPS spectra, stating that the earlier used fitting parameters are incorrect, as a much wider shift between the Sn^2+^ and Sn^4+^ is expected. Consequently, instead of the formation of stoichiometric SnO and SnO_2_, the observed spectra should be interpreted as intermediate oxides SnO_*x*_ and SnO_*y*_ where 1 < *x* < *y* < 2.

Tchaplyguine et al. (2019)^54^ suggested new fitting parameters to include the intermediate oxides in the peak fitting and an energy shift of 1.3 eV between zerovalent Sn and SnO. SnO_*x*_ should be shifted 1.6 eV and SnO_*y*_ 2.5 eV higher with respect to zerovalent Sn, and finally, SnO_2_ should have a binding energy 4 eV higher than the zerovalent Sn. This contrasts with the suggestion of De Padova et al. (1994)^36^ who fitted SnO_2_ at a binding energy 2 eV higher than the zerovalent Sn.

The two alternative fitting models presented by De Padova et al. (1994)^36^ and Tchaplyguine et al. (2019)^54^ have both been used to fit the experimental data acquired in this study. From the results, it cannot be determined which of these models is the most accurate. The two different fitting models adequately reproduced the experimental data, and neither of them stood out as a superior representation of the data (Figure S2). As a matter of fact, by comparing the relative peak areas of the different components for the two different peak models (Table 1), in most cases, the intermediate oxides from the Tchaplyguine model are in practice interchangeable with the stoichiometric oxides from the De Padova model. SnO_*x*_ followed the same trend as SnO, and SnO_*y*_ followed the same trend as SnO_2_. Only at the highest temperature (900 °C) does the contribution of SnO become significant and considerably influences peak fitting. Most importantly, using one or the other assumption doing the XPS fitting does not change our trends observed.

**Table 1:** Comparison between Stoichiometric and Intermediate Oxides Peak Fitting Models for the Sn Nanoparticles Generated at 5% H_2_ (Figure 4a)^a^

	relative peak area (%)							
	De Padova et al. (1994)^36^			Tchaplyguine et al. (2019)^54^				
temperature (°C)	Sn(0)	SnO	SnO_2_	Sn(0)	SnO	SnO_*x*_	SnO_*y*_	SnO_2_
300	0	56	44	0	5	63	32	0
400	0	57	43	0	0	68	32	0
450	0	61	39	0	5	68	27	0
500	0	59	41	0	0	68	32	0
550	0	62	38	0	3	70	27	0
600	0	69	31	0	0	78	22	0
650	0	69	31	0	0	77	23	0
700	0	86	14	1	0	88	11	0
750	0	94	6	0	0	94	6	0
800	0	91	9	0	0	93	7	0
850	2	89	9	3	0	89	8	0
900	8	67	25	8	35	41	16	0

a Values represent relative peak areas in %.

The models of De Padova et al. (1994)^36^ and Tchaplyguine et al. (2019)^54^ provide acceptable fitting of the experimental data, yet they suggest two completely different surface oxidations. The data presented here do not indicate any presence of the formation of SnO_2_ if they are interpreted using the model of Tchaplyguine et al. (2019),^54^ i.e., shifted 4 eV to higher energy compared to Sn^0^. Because of this reason, we chose to present our main findings using the De Padova model. However, our experiments attempted to reduce the surface composition and not to oxidize it. It is therefore possible that only intermediate oxides—and not the SnO_2_—are formed on the surface.

### 3.4 Electronic Work Function

In the results presented to this point, we have primarily based our findings on the changes in the relative peak areas between the different Sn 4d doublets. However, looking into the details, some shifts in the fitted energies of the peaks are also observed. As presented, the observed shifts varied with the external conditions, such as the composition of the carrier gas, annealing temperature, size selection, and alloying elements. All peak fitting parameters, (fwhm, spin–orbit splitting of the doublets, energy difference between the doublets corresponding to different oxidation states, and peak shape) were kept constant, except for the peak intensities and the position of the Sn^4+^_5/2_ peak that was kept as a free variable. As a result, any shift in the fitted energy of the Sn^4+^_5/2_ peak induced a shift in all other peaks since the energy difference between the doublets and oxidation states was kept as fixed variables.

The observed shift in peak energies is attributed to the change in the electronic work function of the Sn particles. During in-flight XPS, the binding energy of the electrons is measured relative to the vacuum level and not the Fermi level. In other words, the binding energy here is determined by subtracting the measured kinetic energy of the electrons from the photon energy. The binding energies in these measurements are therefore defined as the sum of the materials’ binding energy and the work function. The electronic work function is a surface specific parameter that is known to depend on the chemical composition of the particles at the surface. The work function of aerosol particles has previously been studied in-flight by photoemission spectroscopy,^55,56^ and the results have further been used to estimate changes in the chemical composition.

In our investigation, we can simultaneously probe the surface oxidation of the particles and relate it to changes in the electronic work function of the particles. The electronic work function was calculated by subtracting the established binding energy of zerovalent Sn (24 eV) with the fitted binding energy from the experimental data. The calculated work function is compared with an estimated weighted work function based on the surface oxidation state obtained from the results displayed in Figures 2–4. This estimation is based on the reference values for electronic work function of Sn and Sn-oxides (4.38 eV for Sn^0^, 5.2 eV for Sn^2+^, and 4.84 eV for Sn^4+^).^57,58^

**Figure 5:**
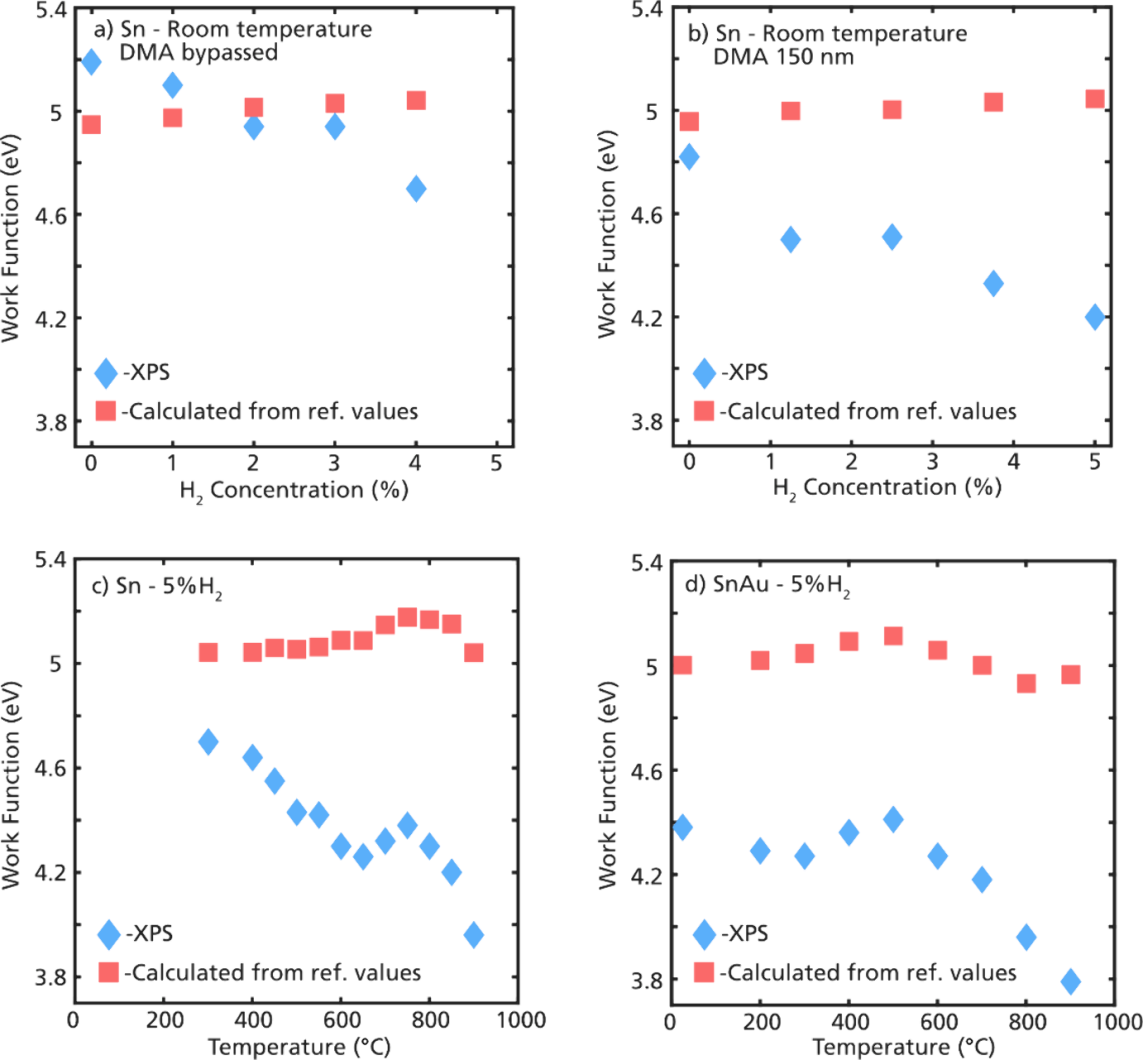
The electronic work function is plotted for different particle generation conditions. In panels a and b, the work function of either full-size (a) or size-selected (b) distribution is plotted against different amounts of H_2_ in the carrier gas but at room temperature. In panels c and d, the work function is plotted against the in-flight heating temperatures for Sn (c) and SnAu (d) with a carrier gas of N_2_ and 5% H_2_. The blue diamonds are the data extracted from the peak positions in Figures 2 and 4, and the red squares are calculated using the relative peak areas of the different Sn 4d doublets and reference values for the work functions.

In Figure 5, the calculated electronic work function is plotted as a function of the H_2_ concentration and in-flight heating temperature. It can be observed that the electronic work function of the aerosol nanoparticles varies substantially with the temperature and with the H_2_ concentration in the carrier gas. The red squares in panels a–d show the expected change in work function when only the change in surface oxidation is considered and using the reference values stated above. However, the change in work function that is observed from the free-flying particles is much larger. The measured electronic work function of the nanoparticles displays a steady decrease as the H_2_ concentration increases (Figure 5a,b, blue diamonds), while the expected work function based on the chemical surface composition should be rather constant based on calculations, showing only a slight increase with increasing H_2_ concentration (Figure 5a,b, red squares). H_2_ is known to adsorb onto the SnO_2_ surface, typically with the formation of water,^59^ and the chemisorption of H_2_ on the surface is expected to lower the electronic work function.^40^ This provides a likely explanation for the observed decrease in the work function with the increasing amount of H_2_ in the carrier gas.

The decrease in work function with increasing H_2_ concentration can be observed for both the size-selected aerosol and the full-size distribution; however, there is one discrepancy. For the size-selected agglomerates, the work function was approximately 0.3–0.4 eV lower (Figure 5b) compared to the full-size distribution (Figure 5a). This can be explained by the different charge distribution of the agglomerates. The agglomerates in Figure 5b were size-selected in-flight by DMA, which operates by selecting particles of a certain electrical mobility. The DMA was set to positive polarity, selecting only negatively charged particles. The particles from the full-size distribution on the other hand carried a known and close to neutral charge distribution after passing through a bipolar charger before the XPS measurement.^31^ It requires less energy for electrons to be emitted from negatively charged particles than from neutral or positively charged particles, which would explain the lower work function for the size-selected agglomerates.

The heated Sn and Sn–Au nanoparticles (Figure 5c,d) revealed a drop in work function with increasing temperature, and in addition, a local maximum in the electronic work function was observed. For Sn, this local maximum is observed at 750 °C, while for Sn–Au nanoparticles, it is observed at 500 °C. This is in good agreement with the observed local maximum of Sn^2+^ shown in Figure 4, indicating a relation between the changes in the surface oxidation and work function.

There are many additional factors apart from surface composition that also influence the work function of aerosol particles. A continuous decline in work function with increasing in-flight temperature has been reported previously,^55,60,61^ with several possible explanations. Smaller particles will have a slightly higher work function than bulk, which depends on particle size.^41^ By increasing the annealing temperatures, the primary particles will grow in size, which would result in a lower work function. The work function with respect to particle size changes with 1.08/*D*(*nm*)^41,56^ and becomes pronounced for sub-10 nm particles. From Figure 3 (top row), it is evident that the primary particle size of the agglomerates increases with increasing temperature. The many primary particles visible at 300 °C have different sizes, but most particles have a diameter of approximately 5 nm. In contrast, at 700 °C, the particles in the agglomerate have a diameter of approximately 70 nm. Based on the above-mentioned relation, this would approximately decrease the work function by not more than 0.2 eV when increasing the temperature from 300 to 700 °C. Changes in the primary particle size can therefore not be the only explanation for the change in work function with increasing temperatures. Another possible explanation could be the changes in charge distribution expected from induced thermal charging^62^ or an increase in the amount of chemisorbed gas molecules.^55^

## Conclusions

This study demonstrates how the surface oxidation of free-flying Sn metal oxide nanoparticles can be varied by changing the gas-phase generation parameters in-flight. We also showcase how a novel setup, available at the MAX IV synchrotron facility, can be used to study the surface oxidation of aerosol nanoparticles in-flight, just seconds after generation, using XPS. We conducted a comprehensive analysis of the surface oxidation behavior of aerosolized Sn nanoparticles under different generation conditions.

Our results show that the surface of these Sn nanoparticles is highly oxidized and that a significant reduction of the surface oxidation is achieved by increasing the H_2_ concentration in the gas and the temperature during in-flight heating. We provide key insights into the surface oxidation process, showing that modifying carrier gas composition, introducing mixing elements, and applying heat to the aerosol particles will change the surface oxide dramatically. We further demonstrate that zerovalent Sn is formed at the nanoparticle surface if heated in a reducing carrier gas. Understanding the surface oxidation of Sn metal oxide nanoparticles is essential for optimizing the development of Sn-based gas sensors and catalytic nanoparticles.

Additionally, this study highlights that surface oxidation directly correlates with changes in electronic work function but also that other factors, such as carrier gas composition, charge state, and annealing temperature, also influence work function in-flight during gas-phase generation. Overall, this work and the methodology employed offer valuable knowledge for the development of engineered metal oxide nanoparticles, contributing to advancements in nanoparticle design and application.

## Supporting Information Available

Sn 4d experimental photoelectron spectra normalized to measuring time, before and after background subtraction (Figure S1), and photoelectron spectra for Sn nanoparticles and comparison between different fitting model (Figure S2) (PDF)
