# Correction to “In-Flight Observation and Surface Oxidation Modification of Tin Oxide Nanoparticles for Gas Sensing Applications”

**DOI:** 10.1021/acsanm.6c03056

**Published:** 2026-07-21

**Authors:** Calle Preger, Linnéa Jönsson, Pau Ternero, Mehran Sedrpooshan, Marie Bermeo Vargas, Antti Kivimäki, Noelle Walsh, Maria E. Messing, Axel Christian Eriksson, Jenny Rissler

In our original article, Figure 4b includes four Sn–Au spectra at 300, 500, 700, and 900 °C that were previously presented in Figure 5 in Ternero et al. (2024)^1^ This prior publication was not clearly acknowledged in the original publication. In Ternero et al., these spectra were discussed in the context of particle bulk structure, whereas in our article we included additional spectra at various temperatures to examine the evolution of surface oxidation with respect to Sn. The article has now been revised to properly acknowledge the original source.

In Section 3.2, fifth paragraph the following sentence has been modified: *The change in elemental bulk ratio along with detailed crystalline structure of these Sn–Au nanoparticles* **together with in-flight XPS for selected temperatures have been** *presented elsewhere* ^45^. In the sixth paragraph the following have been added: *The XPS spectra of Sn–Au at 300, 500, 700, and 900 °C have been presented previously in Figure 5 in Ternero et al. (2024) and were discussed with respect to particle bulk structure* ^45^. *Here additional spectra at various temperatures are added to compare the surface oxidation evolution with respect to Sn.* Finally, the following have been added to the caption in Figure 4: *The spectra in (b) at 300, 500, 700, and 900 °C has previously been published in Figure 5 in Ternero et al. (2004)* ^45^, *Copyright © 2024 The Authors. Published by American Chemical Society.*

These corrections do not affect the conclusions of the work. We apologize for any inconvenience caused to the readers.
