# Supplementary material for: In-Flight Observation and Surface Oxidation Modification of Tin Oxide Nanoparticles for Gas Sensing Applications
Source: ACS Appl Nano Mater. 2025 Mar 18;8(12):6004–13. doi: 10.1021/acsanm.5c00144 (PMC11959520; doi:10.1021/acsanm.5c00144)
Supplement: Supplementary file 1 — an5c00144_si_001.pdf [file an5c00144_si_001.pdf]

Supporting Information:
In-flight Observation and Surface Oxidation Modification of Tin Oxide Nanoparticles for Gas Sensing Applications

Calle Preger*[1,2,3], Linnéa Jönsson[3,4], Pau Ternero[3,4], Mehran Sedrpooshan[3,5], Marie Bermeo Vargas[3,4], Antti Kivimäki[1], Noelle Walsh[1], Maria E. Messing[3,4], Axel Christian Eriksson[2,3], Jenny Rissler*[2,3,6]

1: MAX IV Laboratory, Lund University, Box 118, 221 00 Lund, Sweden
2: Ergonomics and Aerosol Technology, Lund University, Box 118, 221 00 Lund, Sweden
3: NanoLund, Lund University, Box 118, 221 00 Lund, Sweden
4: Solid State Physics, Lund University, Box 118, 221 00 Lund, Sweden
5: Synchrotron Radiation Research, Lund University, Box 118, 221 00 Lund, Sweden
6: RISE Research Institutes of Sweden, Scheelevägen 17, 223 70 Lund, Sweden
*Split corresponding authorship: calle.preger@maxiv.lu.se, jenny.rissler@design.lth.se

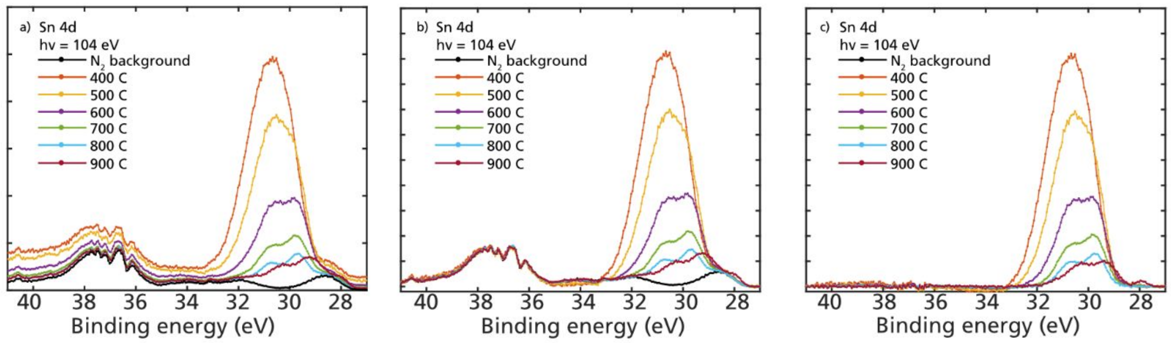

Figure S1 Photoelectron spectra for Sn 4d generated with a carrier gas with 5 % H_2 at different in-flight heating temperatures. (a) Experimental photoelectron spectra normalized to measuring time. (b) Same spectra as in (a) but after Tougaard background subtracted. (c) same spectra as in (b) but after background gas contribution subtraction.

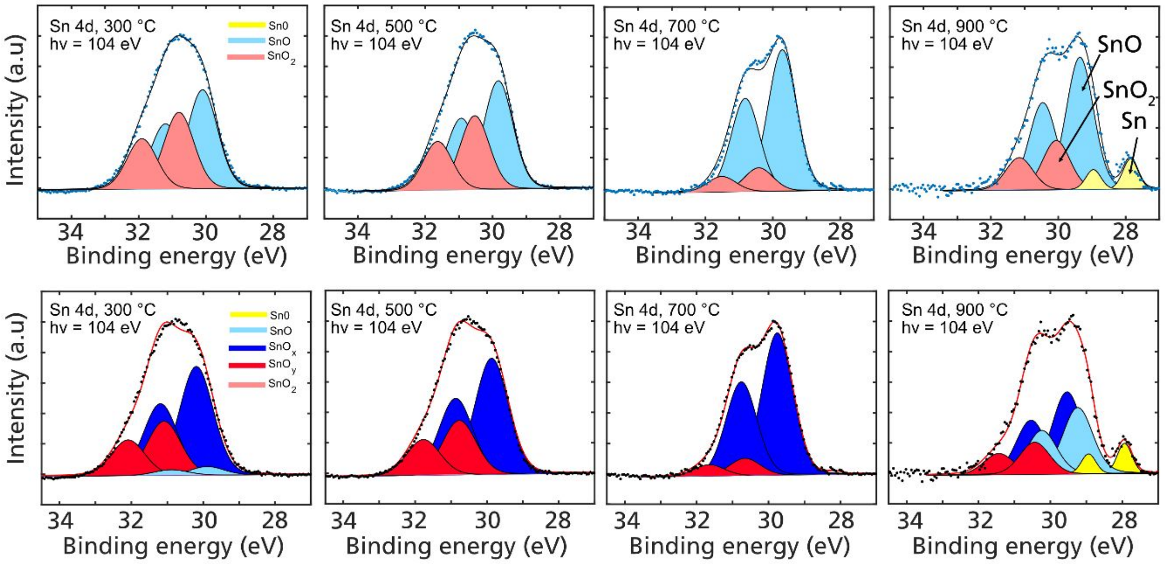

Figure S2. Photoelectron spectra for Sn nanoparticles generated with 5% H_2 at different temperature. Comparison between Padova[1] (top row) and Tchaplyguine[2] (bottom row) model fits.

References

(1) De Padova, P.; Fanfoni, M.; Larciprete, R.; Mangiantini, M.; Priori, S.; Perfetti, P. A Synchrotron Radiation Photoemission Study of the Oxidation of Tin. *Surf. Sci.* **1994**, *313* (3), 379–391. https://doi.org/10.1016/0039-6028(94)90058-2.

(2) Tchaplyguine, M.; Wright, C.; Shavorskiy, A.; Zhu, S.; Mikkelä, M. H.; Zhang, C.; Björneholm, O.; Mårsell, E.; Mikkelsen, A.; Sorensen, S.; Hetherington, C. J. D.; Wallenberg, L. R. Tin-Oxide Nanoparticles Deposited from a Beam: What Happens to the Composition? *Phys. Chem. Chem. Phys.* **2019**, *21* (11), 6287–6295. https://doi.org/10.1039/c8cp06168h.
